# Continuity and change?: Exploring reactions to a guided self-management intervention in a randomised controlled trial for IBS with reference to prior experience of managing a long term condition

**DOI:** 10.1186/1745-6215-8-6

**Published:** 2007-02-22

**Authors:** Anne Rogers, Victoria Lee, Anne Kennedy

**Affiliations:** 1 National Primary Care Research and Development Centre, 5^th ^floor Williamson Building, The University of Manchester, Oxford Road, Manchester, 13 9PL, UK

## Abstract

Self-care interventions are promoted as effective strategies for improving the quality of life and health outcomes for individuals with long-term health conditions. Outcome measures used in evaluations using Randomised Controlled Trials (RCTs) are not designed to consider patients' prior management strategies and experience of illness. Yet the experience of illness literature suggests that adjusting to living with chronic illness, together with broader contextual influences, are likely to be relevant to understanding responses to self-management initiatives. Using group and individual interview data we attempt to illuminate the transposition of IBS from a condition unsatisfactorily managed by medicine to one successfully managed within the life worlds of individuals. If routine embedding of complex interventions depends on the accomplishment of integration and workability in patients' everyday lives then the design and evaluation of such interventions should view participation as part of a process of continuity as well as change. Responses to formal self-management can be extended beyond psychological and other quantitatively measured outcomes. A useful addendum to trial outcomes for self-management education is an understanding of change as being inextricably linked to people's previous attempts to, and experience of, managing long-term conditions. We suggest that the benefits of understanding the prior experience of managing illness and contact with health services include the acceptability and workability of complex interventions in patients' everyday lives.

## Background

### Introduction

Self-care interventions which have been evaluated using RCTs are currently being promoted as an effective strategy for improving quality of life, health and utilisation outcomes for individuals suffering from a range of long-term health conditions. Techniques such as cognitive re-structuring form the bases of self-management interventions and dedicated self-care education training has been attributed with beneficial outcomes including improving people's confidence in their ability to take care of themselves and engagement in collaborative shared decision making with health professionals [1-4]. The success of these interventions has been predicated in large on outcomes relating to: changes in behavior (the changing of diet, relaxation, and 'planned activity'); decreased utilization of health services; and attitude (self efficacy) or being better able to 'cope with symptoms'.

Behaviorally orientated self-management educational programmes work include reference to stages, critical periods, developmental tasks and notions of maturation. Psychological outcome measures such as 'self-efficacy' are viewed as being enhanced through a number of mechanisms, the most effective of which is 'performance attainment' (i.e. actual experience of the success of actions) whilst depression and anxiety are viewed as impairing both self-efficacy beliefs and the ability to engage in those behaviours that might increase self-efficacy [5].

Individuals entering self-management programmes as part of an RCT are viewed as having the potential to self-manage and learn new 'skills' once they have followed a formulaic. However, RCTs are not usually designed in a way which enables the distinction of the effects of past experience when testing new interventions and there is a tendency to treat the person as a blank sheet as far as prior patient experience and management of a long term condition is concerned. We know from the sociological literature on the experience of chronic illness that disruption to identity, personal and social adjustments, and illness management are salient, integral parts of living with a long term condition [6,7].

This literature has pointed out ways in which people adapt, change and develop strategies for managing chronic illness as a naturalistic response to a chronic illness diagnosis. However, we have little understanding of what happens to people's management of their condition when methods designed to change illness behavior or improve knowledge are introduced. Thus, the purpose of this paper is to explore the nature of change that results from being exposed to a guided self-management intervention run as an RCT, from a starting point which considers the way in which people have previously learned to live with, adapt and seek help for a chronic condition – in this instance irritable bowel syndrome (IBS).

Using interviews with IBS sufferers, this paper adopts an approach which explores participants' reactions to a formal self-management programme (as part of a complex intervention), with reference to the way in which health actions and responses may change as a result of engagement with technology, prior personal management strategies and contact and engagement with medicine.

### The sociology of chronic illness: methods of managing and contact with services

The viability of understanding illness experience and management involves a recursive relationship in which service contact reinforces or changes illness identities and illness-related activities. Being diagnosed with a chronic illness involves disruption to the normal life course [6], changes to self-perception, adaptation to the social world, the re-definition of people's competence as social actors [8] and the protection of self-identity from the threat of stigma. The parameters of change from this perspective can be viewed as a search for meaning and legitimacy following the onset or labelling of a chronic condition and attempts made to negotiate a new personal and social equilibrium. An essential part of this process necessarily draws upon the various coping mechanisms, strategies and styles of adjustment which individuals develop over time [9].

From the vantage point of responding to the disruption of chronic illness, 'coping' refers to a sense of coherence which individuals are able to maintain in the face of their condition (for example, a frequently identified response to chronic illness is the way in which people strive to preserve a semblance of 'normality' to the outside world). Self-management is represented by the mobilization of resources and the maintenance of normal activities and relationships (family, friends and occupations) in the face of an altered situation[6]. The notion of 'strategy' within the sociological literature captures the practical steps taken in order to mobilize resources and minimize problems in everyday life[10] and 'style', has been used to denote some of the symbolic ways in which people respond to, and present, their illness, both to themselves and others[11]. The impact of illness on the self has been conceptualized as a biographical disruption, and biographical work in the form of construction and reconstruction persists as part of the continuation of illness. A number of studies have focused on identity work, for example in viewing illness as a vehicle for personal transformation through self-change narratives[12].

### A recursive relationship with health services and technology

Clinical settings and the use of technologies in everyday routines for managing chronic illness can also be viewed as arenas in which problems about the management of illness are constituted and alternative approaches and solutions are pursued and reconfigured between professionals and their patients[13]

The notion of 'turning points' (moments of a radical change in the expected course of a person's career) which people can reach by different routes[14] may signify changes in personal management. A turning point can, for example, constitute a moment when people no longer agree with an approach adopted by the health care system, or a re-appraisal of the meaning of past experiences which enables new insights and permits the taking of an active standpoint. It has been suggested that turning points may occur for people with long term conditions such as chronic back pain after hope has been abandoned of finding understanding and treatment within mainstream health services and disappointments have led to a 'spoiled identity'[15]. This latter observation points to the significance of viewing self-management as a continuum rather than separated out from people's contact with health services.

The role played by service contact has at times been considered as something separated off from people's own efforts to manage illness. For example, self-management has been viewed as purposive action which stops at the outset of the consultation in which 'proper' medicine takes over [16] or something which is marginalized in the consultation in favor of medical instruction[17]. This picture of a 'failure' of medicine to engage with the life worlds of patients has encouraged a view in which living with chronic illness is conceptualized as a reactive flight into normalization in which over time the patient becomes 'free of medicine'. However, others have viewed clinical settings as more connected to the accessing of resources to self-manage and people's illness management strategies. Contact with a variety of resources, services and expert knowledge, have been viewed as invoking a process whereby individuals bring into play frequent internal contestation and revision of what constitutes legitimate expert knowledge about the best way to manage health and illness[18].

We also know that the health actions are established or modified by the interactions that take place between individuals and professionals in health service settings [19-21]. Thus, theoretically, the success or otherwise of an intervention designed to promote or support self-management is likely to be influenced by the extent to which services are able to continue to find solutions to symptoms.

### IBS as a condition

In the medical literature the term 'functional bowel disorder' describes an array of symptoms including abdominal pain and disturbed bowel function without any obvious underlying pathological cause. Criteria have been applied to define categories of functional bowel disorders such as the Rome II diagnostic criteria for IBS[22].

It is not uncertainty over diagnosis, but a lack of definite means of management or symptom reduction, which demarcates IBS from other chronic conditions. Both medical practitioners and patients experience frustration with IBS. For the former this is seemingly due to the shortage of effective interventions. Patients tend to find their IBS symptoms physically troublesome and can be frustrated by a response implying a psychological cause[23,24].

This qualitative study was undertaken to explore any 'change' that might underlie an intervention designed to promote self-care for IBS with reference to a prior sense of self in living with an illness that is neither classified as chronic or acute. We also explore the ways in which people have previously managed as a naturalistic response to having been diagnosed with IBS and their contacts and relationships with health services.

## Methods

A description of the methods and main findings from the accompanying randomised controlled trial are given below:

### Details of the randomised controlled trail

As part of a large randomised controlled trial of self-management interventions in patients with a primary care diagnosis of irritable bowel syndrome (IBS), 458 consecutive patients were identified by their primary care physician if they fulfilled the following criteria: a primary care diagnosis of IBS; two or more consultations for IBS in the year prior to recruitment; and ability to read English. 420 participants from 54 primary care centres agreed to participate in the trial and were randomly allocated to one of three groups, which were stratified according to duration of illness, frequency of primary care visits, age and gender[25]. All participating patients provided written informed consent and the study was approved by the relevant Local Research Ethics Committees.

Patients in the first intervention group received a comprehensive self-help guidebook (produced following a series of earlier focus-group meetings with other IBS patients who described the information they required to help them cope with their symptoms better[26]. The guidebook contained information about lifestyle, diet, pharmacological and alternative therapies and was based on up-to-date evidence-based information and patients' own anecdotal experiences). Patients in the second intervention group were given the guidebook and invited to participate in a one-off self-help group meeting (4–9 patients) facilitated by the trial co-ordinator. The rationale for holding these meetings was that people would benefit from the mutual support. A total of 10, separate, meetings were conducted in different localities around Greater Manchester at least one month after participants had been recruited to the trial. Each session was scheduled for two hours during which patients shared their experiences of living with functional bowel symptoms and described approaches which helped them to manage their illness. Sessions were audio-taped and transcripts were produced. Patients in the control group continued to receive their usual care at the discretion of the primary care physician, receiving the guidebook only at the end of the trial. Patients in all groups could visit their primary care physician without restriction.

Data were collected using self-completed questionnaires and primary care records.

Patients in the intervention groups that received the IBS guidebook had a 60% reduction in primary care consultations (p < 0.001) and a reduction in perceived symptom severity (p < 0.001) compared with controls.

A total of 420 patients from 54 primary care centres were randomised either to receive self-help information in the form of a guidebook or the guidebook plus a "self-help" group meeting or to be in a control group receiving neither intervention. Data were collected using questionnaires and primary care records. RESULTS: At one year, patients in the guidebook group had a 60% reduction in primary care consultations (p < 0.001) and a reduction in perceived symptom severity (p < 0.001) compared with controls. Allocation to the self-help group conferred no additional benefit. Actual symptom scores did not change significantly in any group. Costs per patient were reduced by £73 (confidence interval £43, £103) or 40% per year[25].

### Qualitative methods

The main purpose of the qualitative analysis was to compare and contrast the ways in which people conceptualized their previous illness experience, treatment and management of IBS, with perceived changes as a result of the self-management intervention. Qualitative data resulted from 2 main sources – 'self-help' group meetings and post-trial semi-structured interviews, all of which were audio-taped and transcripts were produced.

Self-help group meetings: Participants in one of the two intervention groups were given the IBS guidebook and also invited to attend a one-off self-help group meeting with fellow IBS sufferers (see page 11). The rationale for holding these meetings was that people would benefit from the mutual support. A total of 10, separate, meetings were conducted in different localities around Greater Manchester at least one month after participants had been recruited to the trial. Although 146 participants were randomly assigned to this intervention group, only 59 actually attended a meeting. This obviously introduces an unintentional bias to the data collection, which unfortunately was out of our control (see Table 1 for demographics relating to Self-Help Group meetings).

**Table 1:** Details of self-help group meetings

Number of meetings held	10
Number attended/number invited	59/146
Attendance per group (range)	4 to 9
Number of female attendees (%)	53 (90%)
Age range years (mean)	22–6 (45)
Years since diagnosis (mean)	0–31 (6)
In employment (%)	36 (61%)
Educated to degree level (%)	17 (29%)

At the end of the year-long trial, the trial co-ordinator conducted semi-structured interviews with a purposefully selected group of 12 trial participants, spanning all trial groups – four with participants who had received only the IBS guidebook, four with participants who had both received the guidebook and had been invited to attend a self-help group meeting and indeed did so, one with a guidebook receiver but a self-help group meeting non-attender, and three interviews with control group participants, who actually received the IBS guidebook at the end of the interview (see Table 2 for patient details and demographics).

**Table 2:** Qualitative Interview Patient Details

ID	Group	Age	Length of time (years) since IBS diagnosed (recorded at time of recruitment to trial)	Working or not	Degree or not
74	2	30	<1	y	y
116	1	36	11	y	n
131	2	58	16	n	n
182	2*	37	6	y	y
191	1	52	10	y	n
198	2	76	31	y	y
235	1	30	2	y	y
266	3	47	5	y	y
274	3	34	<1	y	y
332	3	51	<1	y	n
338	1	57	<1	n	y
355	2	30	12	y	n

* Non-attender

Interview schedule for face-to-face post-trial interviews – Focusing on Pre- and Post Intervention Strategies

• Remind me how long you have suffered with IBS

• Expectations about being on the trial – what did you think the research was going to be about? Were you happy about being the group that you were randomly assigned to? Has the trial lived up to your expectations?

• Management of IBS symptoms before and after being in trial

• Have your ideas about IBS changed by being on this trial?

If so how and why?

• Before coming on this trial what did you think caused your IBS?

(e.g. stress, psychological, etc)

What do you think causes it now?

• Psychological impact of IBS

• Impact of IBS on family and social life – quality of life type issues

• General use of health care resources before trial

Has their use of health care resources changed over the course of the year?

(e.g. GP, secondary care, pharmacist, alternative practitioner, health food shop)

If not seeing GP now – why? Is it lack of support on behalf of GP, or don't feel the need? What would make you go back and see GP more or seek other professional advice?

If like treatments – why? What is it about alternative medicine that is more acceptable than prescribed medicine?

Views about alternative therapy (what's going on – thought processes

why that route?

• Has being in study changed the way in which you interact with your GP?

If so, how?

• Use of Guidebook

What did you find personally relevant?

Did you use it?

Frequency of use?

Was it helpful?

Has it given you confidence?

Did you try any treatments mentioned in it?

Was there anything else that you think should have been included?

• For Patients assigned to Guidebook + Self-help Group Meeting intervention

Did you attend Self-help Group Meeting?

Did you find it helpful? If so, what did you find helpful?

Did you learn anything new?

Would you attend another meeting of this type?

Have you met up with any of the other attendees?

Themes that emerged from both the individual and group interviews were similar. However, it was clear that the lay strategies and opinions in the self-help group meetings were more vociferously expressed. This is consistent with the literature on focus groups which suggests that the bonds of solidarity between people shape narratives of illness and its management through identification with others. The narratives of experience contained in the guidebook resonated authentically with people's shared everyday lives. Whilst the self-help groups illuminated issues that were more nascent or tentatively expressed in the post-trial face-to-face interviews, the nature of the themes were similar, so the data has been treated in a similar way as far as thematic analysis is concerned.

### Analysis

The transcripts were read by all the researchers and the transcripts from the semi-structured interviews and the self-help group meetings were analysed thematically. All interviews were audio-taped with permission and then transcribed. Interviews lasted between 40 minutes and 90 minutes. Analysis began soon after each interview was transcribed and themes identified from the data. Analysis was carried out by the all authors. Each transcript was analysed for its main themes and themes were arranged in categories and the transcripts were analysed against one another by constant comparison[26].

Excerpts of transcribed data were cut and pasted into the categories. Deviant cases were actively sought (for example through identifying and interviewing people who reported no change or scored negatively on outcome measures). The key themes included: the lived experience of IBS (impact on everyday life, experience of symptoms, and reaction of others) ways of managing, lay epidemiology, experience of medical management and diagnosis, alternative help-seeking views about medication, the guidebook as projected identification with others, use of the guidebook, together with perceived changes and continuity from being part of the trial.

## Results

### Experiencing and managing irritable bowel syndrome prior to using self-management

The baseline for 'change' is viewed here as the way in which people managed IBS by themselves and as a result of being in contact with services. People described IBS as a physical and chronic condition, the consequences of which increased over time. What started as a relatively minor complaint became to the IBS sufferer more significant over time and was compounded by an increasing sense of a personal lack of control over symptoms and their management:

'I think it's quite... it's more debilitating than you thought of at first. I think at first I thought Well this is a damn nuisance", but then when you've had it a lot of times, it really, it is debilitating (laughs)' (ID. 338; Guidebook only; relatively newly diagnosed)

Of central significance was the need to retain a sense of normality and ability to continue an ordinary everyday existence. Strategies for managing and coping with IBS were varied. People had made substantial changes to daily routines, food and eating, and other cognitive strategies as a means of managing symptoms as they arose.

'I used to get very bad, like, as if someone's wringing out the washing in my tummy, bad pains and I just used to take (Buscopan?). And I used to take, just roll up a pillow and stick it on my stomach and lie on it and just, you sort of pass out 'cos it's so painful. ... and it's usually (...) on a morning, usually.' (ID. 182; Guidebook + Self-help Group non-attender; diagnosed some time ago)

'...and if I were going shopping ...I used to think "right there's a toilet there, in Bury there's a toilet there" and any slight little bit of pain we used to edge straight towards the toilet, just in case, and that's how it were, rules, you know, "where's the toilets?" You were frightened of going without a toilet' (ID. 191; Guidebook only; diagnosed some time ago)

'Trial and error. It was very new to me so it was a case of trying things off the shelf, the peppermint tablets and that kind of thing and then really identifying food that triggered it but, more often than not it was stress related so it's very much kind of stress management thing. I just tried everything really.' (ID. 74; Guidebook + Self-help Group attender; newly diagnosed)

People turned to humor as a means of coping with embarrassing symptoms:

'It depends, I mean, obviously ... I would have to, but if I'm in the car with somebody I just say 'Michael, can you open the window right now, 'cos I'm gonna drop one.' (Laughter). I mean, I've done it today, you know. You've just got to laugh about it... A good way to be, you've got to be, haven't you? You've just got to laugh about it 'cos otherwise I'd (run?) myself into the ground getting upset, so you do just make a bit of fun about it. But I do get a bit upset, you know, about my stomach being the way it is, but I just think, well, it could be worse.' (5^th ^Self-Help Group meeting)

Attempts to control symptoms were reported in a way that suggested a sense of precariousness and uncertainty which may have indicated uncertainty about whether what individuals were doing was 'right' or appropriate. The impression given in personal accounts was of the illness having control over the person rather than visa versa.

'Well it sort of rules your life because you can't do the things that you want to do because you're frightened something might happen, like a simple thing like gardening, now I know I love gardening but I know if I'm going to go... because I'm creasing up me stomach and that and then all of a sudden I think Oops and I go and ...' (ID. 191; Guidebook only; diagnosed some time ago)

There was a sense that the nature of the condition was difficult to decipher and solutions, once identified, problematic to enact within the confines of everyday living.

'I just don't understand how you can isolate what it is. I mean, I know I've isolated this lactose just by doing it, going on a milk free diet, but in the myriad of things that happen to you in a day and the myriad of things you eat and drink and the conditions, you know, the conditions you meet each day that you have to deal with, I think it's (very difficult?) to tell what's making you feel ill. (7^th ^Self-Help Group meeting)

### Medical contact: the experience of failing tests and failure to acknowledge suffering

When they were recruited into the trial all of the people we interviewed were in contemporary contact with health services for their IBS. The prospect of medical management held out the hope of gaining an official illness label, validating an illness experience and a new avenue of support from previously having just lived with the problem alone:

'I went to the Well Woman Clinic and they knew about it. And she said to me, "You have IBS, irritable bowel syndrome, and we'll try and help you, and there are a lot of people with it now." And that's how I started to know what was wrong with me.' (ID. 198; Guidebook + Self-help Group meeting attender; diagnosed some time ago)

It also meant that people were sent for complex medical tests. Finding out that experienced pain and discomfort was not something life threatening (such as cancer) came as a relief. However, the help and hope which was seemingly expected was not always realized in people's ongoing contact with services.

Respondents felt that medical practitioners frequently did not consider IBS to be as debilitating and problematic as the respondents. This together with what was viewed as a personal failure on a range of tests left people with a sense of futility, delegitimation and hopelessness about their interactions with health services.

'I've had tests at the hospital. They send you things ... tablets, I'm on ... at the moment ... but when you get a really bad attack or you go back to the doctors... they'll send you for more tests, as if it can't be IBS, it's too bad. So you've got to have this test and that test and the last one (the?) other week and they just... to be quite honest they're barbaric, but to put yourself through that and hope there's something wrong with you, then you know, you know that it's bad, because you're trying to say to the specialist, "Tell me there's something wrong. Please, even if it's really bad." Because you don't want it to be IBS, you want it to be something treatable.' (1^st ^Self-Help Group meeting)

GPs were often reported as resorting to construing IBS symptoms as evidence of a problem of psychogenic origin (particularly when tests failed to reveal a pathological cause). This proved problematic for some. Whilst 'stress' as a triggering factor of symptoms was recognized as being relevant at specific times, the notion of stress as a primary cause resulted in confusion and dissonance when it was deployed in a manner which eclipsed the possibility of other causes and downplayed the embodied experience of having to live with IBS on a daily basis. Emphasizing the psychological without engaging with it in therapeutic terms resulted in people's interpretation that what they were suffering from was not a real disease and that it was not serious. The failure to acknowledge the impact of IBS is indicated in the following quote:

'I mean, that's the worst, one of the things that concerned me, is that I know that I've had IBS for years and years and years, I would say that I'm now in my eleventh or twelfth year, but recognition of the fact, and I did see endless consultants and they've always been very, very careful to say to you, you know, "You're not actually gonna die of this, it doesn't lead to cancer, etc, etc." But what they actually fail to tell you is that actually what you've got is a very serious condition that can actually ruin, and quite often does ruin your life. And nobody ever says that to you, you know. And it was only when I recently saw a young consultant at (X) hospital, and he said to me "Some people with severe IBS it can be just like being on kidney dialysis ... kidney dialysis treatment, it can make you feel in the same way as that kind of patient and can have that kind of severity." And that was like some kind of authentication, because you know, you do think that you're, you know, you know that you're very, very ill and yet nobody ever says to you, "yes", because it's not a clinical condition. And I found that recognition the most helpful thing that I've been told throughout my trials and tribulations. (7^th ^Self-Help Group meeting)

The failure to adequately acknowledge the consequences of the illness for people's everyday lives was compounded by the absence of any effective treatment and management options and abandonment.

'I went to see the doctor, he sent me for an endoscope and it wasn't me stomach and then he sent me for a colonoscopy nothing worn there and the specialist just said "high fiber enjoy your life".' (ID 191; Guidebook only; diagnosed some time ago)

Thus the central dilemma left by prior management of IBS seemed to be an absence of control or advice and acknowledgment of suffering. IBS was viewed as an adversary, which had the upper hand rendering feelings of incompetence and incapacity to manage it. Contact with medicine was seen to reinforce these feelings. There was little on offer in the way of treatment and a seeming absence of engagement with existing activities people themselves were undertaking. This was illuminated in accounts of interactions with GPs when offered the IBS guidebook. A number of people expressed the view that they had come to the end of what limited means of management were on offer, and were receptive to a change which rescued them from a medical cul-de-sac as explained by this participant:

'It was when I went back to my GP and said, "Really I want to come off all this, (medication etc) 'cos I don't think this it's helping." "Oh right there's a self-help book (laughing) available, I'll put you on the research programme". So erm..... I think perhaps it's that idea that we want to help ourselves to start with, that's brought us forward. (9^th ^Self-Help Group meeting)

### The process of change – self-efficacy and beyond

One of the main findings from the RCT was that although actual symptoms did not change, people perceived them as being less severe (see page 11)[25]. Accounts provided by respondents matched the quantitative outcomes identified by the trial. In general, people were planful and anticipated how to control the un-predictive nature of the condition. Regular and frequent references were made to the way in which the individual was better able to 'cope' with and 'control' annoying symptoms. Changes identified included an increase in self-surveillance, monitoring and adjustment to eating routines, together with the avoidance of stressful situations and dietary change:

'I think the main thing I took out of it was more kind of relaxation and stress management, I started to read about that subject rather than irritable bowel itself. Like I said...if I fixate on my symptoms it makes them worse, it's quite a vicious circle really.' (7^th ^Self-Help Group meeting)

'Well I think it's made me actually think about it more really, and I have been... I have been very careful, I've read the book several times and I have watched very carefully what I have eaten and I try not to get into situations where I get stressed out, and I think the combination of that has definitely improved it, I'm not saying it's made it better because I've had a flare-up within the last fortnight, but... and I think I know why I've that flare-up, but the combination of those two things has definitely helped, coupled with the fact that I've started to drink Yakult everyday and I think that's helped as well, so I think that having the booklet and being on the trial has made me think more than I've ever thought about it and I think that's helped.' (ID 131; Guidebook + Self-Help Group attender; diagnosed some time ago)

Whilst the respondents quoted above seemed to have been consciously aware of the psychological means of dealing with matters, there is also an evident emphasis and focus on diet and on tightly managing physical symptoms in people's accounts. This confidence following exposure to the trial seemed to an extent to be rooted in a new found ability to follow self-intuition in managing things the way that people wanted to. The latter seemed to be predicated by an abandonment of medically-orientated ways of managing matters in favor of personal control over symptoms, which was viewed as more reliable. In this sense people seemed to re-construe their previous contact with medicine and process of rejection in order to accept or move on.

'Yeah I don't think you need pills. Right, they give you pills, I don't think you have to have pills every time, any longer, 'cos you would automatically take a pill wouldn't you? Or you can actually have pills all the time can't you, I think? I took that (name of drug) and I didn't like it 'cos I didn't want to take a pill every day for something that might only happen once a month. If I have a really bad attack, if it's just a small one through the day, then I just sit there and breathe it out. I do, you know, just sort of cancel everything out and just go into yourself and I just breathe, like when you're in labor. Just breathe like that and then it usually goes... (ID. 182; Guidebook + Self-Help Group non-attender; diagnosed some time ago)

Of some pertinence to cutting the ties with medicine was a recognition that the setting up of the trial was a form of official acknowledgement and legitimization of their condition:

'...you're in that much of a fog, you don't know what's happening and ... like I came to Doctor C and she said it could be IBS, well the doctor at X had said that too...but it's just a case of, "Well try this, try that", you know for diarrhoea and things like that, "Try these tablets for the pain", but nothing helped, nothing seems to help, it will help for so long and then you seem to get immune to it and it doesn't work no more, so I just thought, "well what's use of keep pestering Doctor C when she doesn't know what to do for me, I don't know what to do for me", and then when I came to see her and she mentioned "Would you go on this trial?", I said, "yes definitely", and ... when I'd seen you, I went home to Stuart and I said, "Do you know I feel right relieved" and he said, "Do you?" and I said, "yes, because at least somebody's there that's trying to get to bottom of it and understand what's happening to you". I feel like somebody's in my corner, fighting for me, and ... I know these things take years and things like that, to find out something definite to help people, but at least you know there's somebody there trying to find something for people.' (ID 191; Guidebook only; diagnosed some time ago)

### The relevance of the Guidebook and lay knowledge to the process of change

Both the content and tangibility of the technology (i.e. the guidebook) had relevance for undertaking new self-management activities. The ready availability of the information was important. Some individuals pointed to the content as being important. However, in some respects the content was not seen as novel. A number of respondents reported receiving information previously but this had not been construed as being particularly helpful:

'I was given a sheet when I was first diagnosed in hospital, so ..., and it was literally like, don't eat everything, don't eat anything ... (laughter) just drink water for the rest of your life. And for the first few weeks I did actually try it and it just didn't make a blind bit of difference, so I just started reintroducing things and then seeing what worked and what didn't work.' (1^st ^Self-Help Group meeting)

The style and presentation of information was compared favorably with the didactic presentation of information from within health services. Thus the book reflected back to people the validity of their own ways of managing and confidence in managing IBS. The lay knowledge contained within the book resonated with what people felt that they had learned themselves over a number of years and acted as a key reference point which provided a point of consolidation and positive reflection on what they had previously undertaken.

'Yeah you feel like you're faking the symptoms don't you, you feel like no one knows how bad it feels, and you're the... and they think you're over-reacting? It's horrible, I've had it for four years, nearly exactly four years, I remember waking up and my stomach being bloated and I found that with the book, although I don't think it's helped me manage my symptoms any more than I already was doing, but that's because over four years I've built up the knowledge, because I've been proactive about finding out things, but if I'd been given that book four year ago it would have been a life-saver.' (10^th ^Self-Help Group meeting)

In a number of the narrative accounts there was some suggestion that medicine and lay expertise were inverted. Individuals' own thoughts about their condition and its management which had previously been of questionable legitimacy were given greater certainty and authority.

'I know you shouldn't self-diagnose, you know, but people do go looking for books and things, and I think from that point of view, if somebody's got all sorts of strange symptoms but hasn't been able to work them out, I think it makes them more successful (at treating their IBS)... (10^th ^Self-Help Group meeting)

Not only did 'the book' represent a systematized way of validating lay knowledge but the proximity of the technology to embodied experience meant that it acted as an instantly accessible source of help which fitted with the episodic nature of IBS.

'...the fact that I had the book to go and look at, even though I wasn't really convinced that it was giving me all the answers, it was something that, it was a starting point really each time I got it. I mean I read it in the first place but then, you know, it's like I had a period of time, I must admit I didn't on the first day think, "Oh I must read the book." It was usually after I'd had three or four really bad days. And I thought I'd best try and do something. Maybe the, you know, maybe I've missed something in the book and then read the book. So yes I did use the book. So, yes it was useful. It's useful to have something in writing. Even though I know in my head all the things that might trigger it off' (ID. 338; Guidebook only; relatively newly diagnosed)

'Well I sat down and read it from front to back when I first got it and then ... if I were doing anything or I fancied eating anything, then I went through book to see what anybody else had said about it, you know, and I thought, "they've tried it and it's done so-and-so, I'll try it and see what happens wi' me" and the if it works same way as what the book said, I thought, "right, that's it", I put a little tick at the side of it as if to say you don't try that again.' (ID. 191; Guidebook only; diagnosed some time ago)

It fitted into the jigsaw and changes of everyday life

'...when it does flare-up I get the book out and I read it and it gets things into perspective again, because you do get things out of perspective, I think, when it's bad ... and I think that I'm beginning to come to terms with the fact that one day you can feel perfectly well and the next day... it comes on so suddenly, and suddenly you feel out of control again, and I think by having the book, reading the book, adjusting the diet, trying not to get into stressful situations, it's all helped, it's all helped. (ID 131; Guidebook + Self-Help Group attender; diagnosed some time ago)

Finally the book helped collectivize (that is, share with others) the experience and management of IBS. In the self-help group meetings it was a clear intention of the design that the process of sharing experiences about IBS and self-management would help construct a group narrative which would provide mutual support. In this respect discussions constituted a symbolic bridge in which attempts were made to repair the dissonance which had arisen between bodily experience and contact and messages received from service contact in primary care. In these group meetings it was clear that the collective narratization enabled individuals to share, reconfigure and resolve through group discussion the alienating aspects of IBS and its management which intruded upon life. However, just as importantly, being able to read the IBS guidebook in the privacy of one's own home also represented the opportunity for people to identify with other people's experiences. The focus on other people's suffering seemed to initiate a form of projected identification in the validation of experience and in efforts made to control IBS.

'A lot of the comments that were made were comments that I could have made myself, I know everybody's different and everybody's symptoms are different and everybody needs different treatment, but I think the comments are common to lots of people with IBS, you all say the same things, so that helped, yes that helped.' (ID 131; Guidebook + Self-Help Group attender; diagnosed some time ago)

A further dimension of the collective use of the book was in providing the means to share knowledge with others and thus permitted a stepping out of an exclusive illness role into one which was of assistance to others.

'...because, you know, everybody you talk to, I mean we have a friend haven't we... she's the opposite way, she's constipated and that's who I passed the book on to, for her to read.' (ID. 191; Guidebook only; diagnosed some time ago)

'It's handy having the booklet because my boyfriend read it and it really helped him, and now he says, "Are you sure you want to eat that? It's got this in", so it's... that's done all my talking for me really, it's a mutual perspective.' (10^th ^Self-Help Group meeting)

## Discussion

Within health services research there is an increasing focus placed on patient response and involvement in complex interventions. (e.g. Featherstone and Donovan)[27]. An important aspect of this is an understanding of the conditions necessary to accommodate and embed complex interventions into the routine elements of the health and illness 'work' undertaken by patients.

Using IBS as an exemplar, this paper has attempted to illuminate the way in which responses to formal self-management can be extended beyond the psychological and other quantitative outcome measures used in trial designs. Repeat qualitative interviews allow processes and change to be captured and examined more accurately and in more detail [28]. Thus one of the limitations of this study was that patients were only interviewed about their prior experiences after their involvement in the trial and therefore accounts were provided with the benefit of hindsight. Thus, describing how things were before the trial might have been influenced by people's subsequent experiences of being in the trial. However, the accounts nonetheless provided in-depth accounts of how people self-managed before the interventions and how individuals thought their coping style and management had changed as a result of being exposed to the IBS guidebook as part of the trial.

We have attempted to view the process of change in self-management as constituting an interactive and continual process with prior ways of managing a condition and contacting services. Notwithstanding the limitations of the study, the analysis suggests that understanding change in self-management benefits from viewing the process as one which can be viewed as part and parcel of people's previous attempts to come to terms with and manage long-term conditions.

The use of qualitative methods were orientated to arriving at more of a contextual view of people's responses to taking part in the intervention which took into account prior experiences of service contact and management in order to illuminate the nature and processes of change for people taking part in a self-management intervention for IBS. In studying the experience and ways of managing prior to and after the trial, the impact of this intervention can be seen in a context which illuminates continuity as well as change in patients' personal long term condition management. Continuity with previous was of managing was evident in the accounts given of the strategies that were used prior to the intervention and afterwards. In terms of change[7] analysis of the accounts showed something about what lay behind people's enhanced confidence to manage their condition better and indicated how the use of the guidebook overcame some of the experienced dissonance people felt about their contact with medical services. In this respect measures of self-efficacy accurately tapped into changes in self-confidence that came about as a result of people being exposed to the self-management programme. Contact with primary and secondary services represented a potential source of diagnosis and assistance but left people with feelings of de-legitimation about their illness and the way in which it was being managed.

It was against this background that the self-management intervention represented an official acknowledgement of suffering and provided a tangible means of the individual accepting 'responsibility' in a way which could be enacted and operationalised on a practical and everyday basis. To cope with IBS and in order to live as normally as possible, people tapped into and reinforced their existing resources and conceptualizations rather than solely inventing new ones. The inclusion of accounts of 'lay' experiences and knowledge in the guidebook was central to this, as was the tangibility and availability of 'just in time' information. Engaging with the contents of the book gave a legitimacy to lay knowledge about the cause as well as management of the condition. Moreover, the book was deployed as a collective resource which was owned and then freely disseminated in people's social networks and beyond, through projective identification and more tangibly in the self-help group meetings. This fed into people's altruistic tendencies as much as being in a group meeting provided mutual support.

The philosophy of self-management promoted in the book, which emphasized a variety of strategies that could be managed and treated IBS as a complex disorder with a range of competing theories about aetiology can be seen as having re-invigorated and resourced patients' attempts to manage their condition.

## Conclusion

Complex interventions such as self-management packages are introduced into the complexity and diversity of patient trajectories which make up the experience of everyday life of living with a chronic condition. The way in which people respond to self-management should thus be seen as part of the narrative disruption and reconstruction which characterizes living with chronic illness and as inextricably linked with the nature of people's engagement with service provision and prior ways of managing health and illness developed over time.

Trial interventions in chronic disease management therefore need to take into account the complexity of the existing ways in which people already manage and have contact with services in explaining change or 'effects'. It has been suggested that the evaluation of complex interventions requires the use of both qualitative and quantitative evidence. In relation to understanding patients' responses to treatment, this study illuminated how prior experience and ways of managing were influenced by exposure to the guidebook, which formed the main focus of a self-management intervention for managing IBS within primary care. The findings of this study highlight the need to include qualitative research into patients' exposure to new treatments which form the bases of complex interventions in order to understand more about the processes and ways in which interventions are accommodated into people's everyday routines and philosophies of illness and service contact.

## Competing interests

The author(s) declare that they have no competing interests.

## Authors' contributions

AR: study design, data analysis, drafted paper; VL main data collection, analysis and contribution to the writing of the paper; AK study design, collected data and contribution to writing the paper.
